# Material Characterization and Substrate Suitability Assessment of Chicken Manure for Dry Batch Anaerobic Digestion Processes

**DOI:** 10.3390/bioengineering7030106

**Published:** 2020-09-07

**Authors:** Harald Wedwitschka, Daniela Gallegos Ibanez, Franziska Schäfer, Earl Jenson, Michael Nelles

**Affiliations:** 1 Department Biochemical Conversion, DBFZ Deutsches Biomasseforschungszentrum gemeinnützige GmbH, Torgauer Straße 116, D-04347 Leipzig, Germany; Daniela.Ibanez@dbfz.de (D.G.I.); Franziska.Schaefer@dbfz.de (F.S.); Michael.Nelles@uni-rostock.de (M.N.); 2 Department Bio Industrial Services, Inno Tech Alberta, PO Bag 4000, HWY 16A and 75 Street, Vegreville, AB T9C 1T4, Canada; Earl.Jenson@InnoTechAlberta.ca; 3 Department Waste and Resource Management, University of Rostock, Justus-von-Liebig Weg 6, D-18057 Rostock, Germany

**Keywords:** dry batch anaerobic digestion, chicken manure, percolation, permeability

## Abstract

Chicken manure is an agricultural residue material with a high biomass potential. The energetical utilization of this feedstock via anaerobic digestion is an interesting waste treatment option. One waste treatment technology most appropriate for the treatment of stackable (non-free-flowing) dry organic waste materials is the dry batch anaerobic digestion process. The aim of this study was to evaluate the substrate suitability of chicken manure from various sources as feedstock for percolation processes. Chicken manure samples from different housing forms were investigated for their chemical and physical material properties, such as feedstock composition, permeability under compaction and material compressibility. The permeability under compaction of chicken manure ranged from impermeable to sufficiently permeable depending on the type of chicken housing, manure age and bedding material used. Porous materials, such as straw and woodchips, were successfully tested as substrate additives with the ability to enhance material mixture properties to yield superior permeability and allow sufficient percolation. In dry anaerobic batch digestion trials at lab scale, the biogas generation of chicken manure with and without any structure material addition was investigated. Digestion trials were carried out without solid inoculum addition and secondary methanization of volatile components. The specific methane yield of dry chicken manure was measured and found to be 120 to 145 mL/g volatile solids (VS) and 70 to 75 mL/g fresh matter (FM), which represents approximately 70% of the methane potential based on fresh mass of common energy crops, such as corn silage.

## 1. Introduction

Due to increasing protein demand for human nutrition, poultry farming is one of the fastest developing sectors of animal husbandry. The FAO stated that, over the last fifty years, poultry was the livestock category with the highest head growth worldwide, with roughly a five-fold increase [1]. In Germany, the total number of chickens in the production cycle increased from 109 million animals in 2003, to approximately 158 million animals in 2016, and the number of chickens kept per farm increased from approximately 1217 to 3361 during this time span [2]. These growing livestock numbers lead to an increase of the process related waste material production. Nebel and Kühne [3] estimated the biomass potential of chicken manure from laying hens to be as much as 5 million t/a plus another 1.3 million t/a from chickens for egg production, pullets and broilers. Dry chicken manure is commonly utilized as agricultural fertilizer, which is rich in nitrogen, potassium, phosphate, calcium, magnesium and sulfur [3]. However, in regions with high livestock numbers, comprehensive manure management is required to reduce the risk of nitrate pollution of surface and ground water and gaseous ammonia (NH_3_) emissions from manure storage and application. According to Haenel et al. [4] the NH_3_ emissions of poultry breeding have a significant share (9%) of the overall NH_3_ emissions from animal husbandry. More than half of these emissions are caused by manure handling

Chicken manure is an agricultural waste material, which is a particularly interesting substrate for biogas production [5]. Compared to other agricultural residue materials that are used as biogas production feedstock, dry chicken manure has a high energy potential based on fresh weight, with an average methane yield of 70–140 m^3^/t. The average methane yields of cattle dung, cattle manure and pig manure are comparably lower with 33–36 m^3^/t, 11–19 m^3^/t and 12–21 m^3^/t [6]. Since chicken manure is an agricultural residue material with a relatively high dry matter content only low production, storage and transportation costs are incurred. Valuable products from the anaerobic digestion process are biomethane, which can serve as a renewable fuel or as an energy source for thermal and electrical energy production, and digestate that can be used as compost, fertilizer or soil amendment. According to Bayrakdar et al. [7] nitrogen extraction from chicken manure digestate for the production of concentrated nitrogen fertilizer has the potential for value added production and reduction of greenhouse gas emissions. In current practice, chicken manure is utilized as a co-substrate by a number of agricultural wet digestion biogas plants. However, the high sand and nitrogen content of the feedstock can lead to technical and biological process problems at full scale, which is why the mono digestion of chicken manure in agricultural biogas plants has proven to be rather challenging [8].

The dry batch anaerobic digestion process is a waste treatment technology most appropriate for the treatment of stackable (non-free-flowing), fibrous and contaminant-containing dry feedstock materials. In contrast to conventional wet digestion systems, these systems rely purely on percolation of process fluid through the feedstock material within a garage type dry digester to facilitate the digestion process [9]. Process fluid is sprayed on top of the substrate and serves as a transport medium for heat, nutrients and material exchange and composes of water and effluent from the digestion process, which is sporadically recycled. Volatile components solubilized in the percolate are simultaneously digested in a separate digester (without or with minimal mixing) that services numerous garage type dry digesters. The feedstock materials are treated in batch operation, which means they are introduced once into the digester, are not actively mixed during the process, and are replaced after a given retention time by fresh substrate. The raw material is generally loaded into the garage type digester along with a fraction of previously digested material (inoculum), which helps to stabilize the process by preventing process acidification [10]. After 20 to 30 days of substrate retention time in the garage type digester, the digested feedstock is replaced with another mixture of fresh and digested material. A further special feature of the process is the discontinuous biogas formation resulting from the batch operation of the process. For this reason, dry batch fermentation plants consist of a series of box fermenters connected in series, which are operated at different batch start and finish times in a rotational cycle to ensure a somewhat constant and consistent biogas formation.

The advantage of this type of dry batch digestion process is a low susceptibility to contaminants, such as sand, stones, glass, plastics or metal, which can lead to an impairment of the stirrer and pump technology in a stirred tank system. Furthermore, the system is relatively unaffected by substances that tend to create sediment layers on the fermenter floor. Since the box system is opened, emptied and recharged after completion of a single fermentation cycle, larger sediment layers on the floor are avoided. In agricultural wet digestion systems large amounts of liquid are required i.e., liquid manure to adjust the water content and viscosity of feedstock mixtures with high dry matter contents. This results in the generation of high volumes of diluted digestate, which increases the costs of digestate storage and transport for agricultural use [11]. In the dry batch digestion process substrate materials can be treated in a stackable form, which results in an efficient utilization of the reactor volume and generation of less liquid digestate [12]. The production of a dry product with minimal wastewater generation makes the dry digestion process appear highly appealing for the treatment of dry chicken manure.

The dry batch anaerobic digestion process requires a feedstock material with sufficient permeable structure that enables percolation of liquids in the anaerobic stage to ensure the mentioned transportation function [13,14]. Insufficient material structure of the input material and insufficient structure stability during the digestion process can lead to a reduced biological degradation if the percolation of the substrate material is uneven or if the percolate accumulates within the digester, which often results in technical problems. Additionally, dry anaerobic batch digestion has to overcome several other challenges. Most dry batch digestion plants use stabilized digestate from previous batches as an inoculum source that needs to be recycled for process stabilization in the case of feedstock with high biodegradability and conversion rate. Consequently, with significant inoculum recycling, the plant capacity has to be larger, which leads to higher investment costs.

The aim of this study was to examine the feedstock suitability of chicken manure for dry batch digestion with regards to material composition and permeability under compaction. Hence, the novelty of this study surrounds oedometer tests that were conducted as a method for material characterization that guided conditioning practices to create an appropriate substrate material structure for dry anaerobic batch digestion of chicken manure at full scale. Furthermore, dry batch anaerobic digestion trials without solid inoculum addition and a secondary methanization digester were carried out with mixtures of chicken manure and different structure material contents. The aim of the digestion trials was to determine the digestibility of chicken manure without inoculation, and to evaluate the influence of structure material addition and material permeability on the specific methane yield. The ultimate goal is to increase the efficiency of percolation processes so that, on the one hand, new plants can enter directly into efficient operation (without long running-in times), and on the other hand, existing plants can optimize their operations and substrate utilization.

## 2. Materials and Methods

Chicken manure samples were tested for their material properties including total solids and volatile solids, nitrogen, protein, fat and fiber composition, permeability under compression and compressibility. Additionally, dry anaerobic batch digestion trials were carried out as a means to evaluate the digestion properties of chicken manure mixtures without inoculum addition and a secondary methanization digester.

### 2.1. Materials

Material characterization tests of chicken manure were carried out with samples collected during 2014 and 2020 from different regions of Saxonia in Germany. Wet sample materials were quickly cooled after sampling and stored at 5 °C before lab analysis. Structure materials such as beech wood chips, wheat straw and plastic carrier (Bioflow 40, RVT Process Equipment GmbH, Steinwiesen, Germany) were provided by the Deutsches Biomasseforschungszentrum gemeinnützige GmbH (DBFZ).

### 2.2. Analytical Methods

A multitude of material characterization tests were performed on chicken manure samples and substrate mixtures of chicken manure after structure material addition. Total solids (TS) and volatile solids (VS) were measured in accordance with DIN EN 12880 (2001) [15] and DIN EN 15935:2012-11 (2012) [16]. The pH-value was measured with a pH device 3310 (WTW Wissenschaftlich-Technische Werkstätten GmbH, Weilheim, Germany). Volatile organic acids, Weender feed analysis, ammonium nitrogen (NH_4_-N) and total ammonia nitrogen (TAN) were determined as described in Liebetrau et al., (2016) [17]. Free ammonia (*FA*) concentrations were calculated according to Equation (1)

(1) FA=0.94412∗NH4/(1 + 10^((0.0925 + (2728,795/(t+273.15)))−pH))

where *NH*_4_ is the total ammonium concentration, *pH* is the *pH*-value and *t* stands for temperature.

### 2.3. Measurement of the Compressibility and Permeability under Compaction

**Figure 1:**
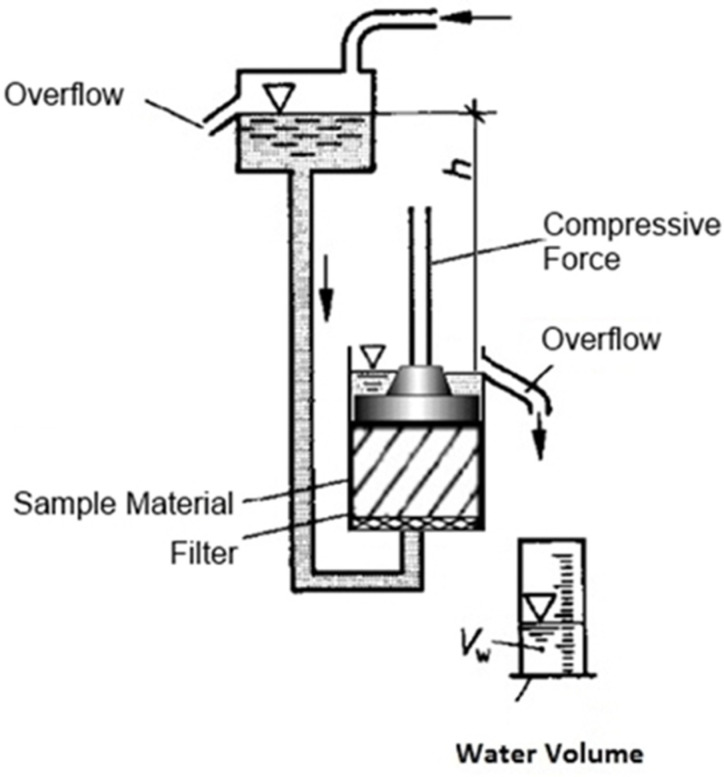
Schematic illustration of the oedometer testing appartus.

In order to determine the influence of the material compaction on substrate permeability and compaction, oedometer tests were conducted to simulate full scale conditions inside a percolation digester. A schematic illustration of the testing stand can be seen in Figure 1. The method development is described in [18]. The height of the stacked substrate within the percolate digester, which can range between 2 and 3 m, results in material compaction and reduced permeability within the substrate heap. An oedometer test apparatus was used to determine the permeability of the sample material under similar material compaction by applying a defined force on the top of the sample, which simulates the compaction that occurs in a full-scale environment with several meters of material height.

**Figure 2:**
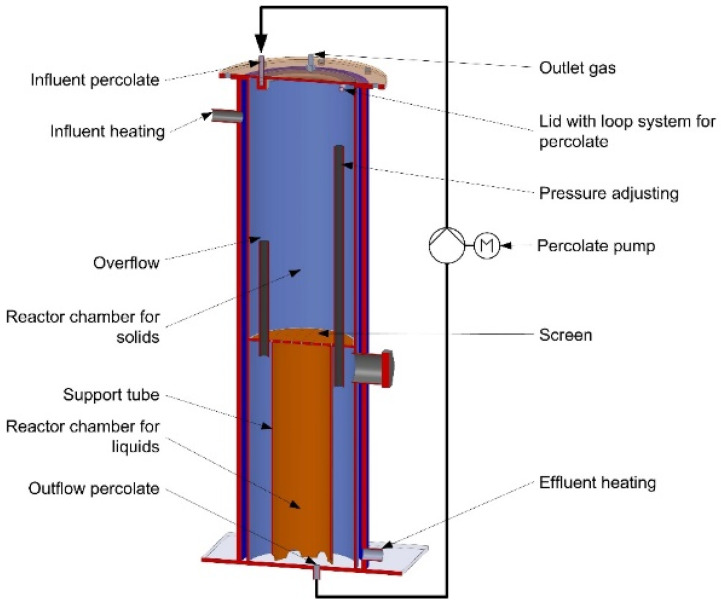
Schematic of percolate digester (dry digestion system) (Stur, Deutsches Biomasseforschungszentrum gGmbH (DBFZ)).

The sample material was placed in an oedometer with a 254 mm diameter and 240 mm height and compressive force was applied with a perforated compression plate with 247 mm diameter. The compression plate was connected to an air piston as seen in Figure 2 that supplied a constant but adjustable force. The hydraulic conductivity and the compressibility of the sample material were determined at loose compaction and low, medium, and high compression equivalent to different material heights encountered in full-scale dry fermentation processes. Hydraulic conductivity first requires measurement of the flow rate *Q* (m^3^/s) through the sample, which is calculated from the volume of water *Vw* (L) passing through the sample material per unit of time *t* (s).

(2) $$Q=\frac{V_{w}}{t}$$

The hydraulic conductivity or permeability *k_f_* (m/s) describes the ease with which the percolate can move through the pore spaces of the sample material. It is calculated by the quotient of the flow rate *Q* (m^3^/s) multiplied by the material sample height *l* (m) divided by the material surface area in the oedometer *A* (m^2^) multiplied by the hydrostatic height *h* (m).

(3) $$k_{f}=\frac{Q*l}{A*h}$$

The simulated material heap height *h_S_* (m) is calculated with the defined force applied by the perforated plate connected to the air piston and the oedometer diameter *d* (m), the density of the water saturated sample *ρ* (kg/m^3^) and the mass *m* (kg) of the sample material describe the simulated material volume. The cumulative compaction or material compressibility is determined by dividing the material height before and after compaction.

(4) $$h_{s}=\frac{4m}{\pi d²\rho }$$

The test setup was configured to measure the material permeability without compaction (*P*1) and with 1.5 m (*P*2) and 3.0 m (*P*3) simulated feedstock heap height. The material compressibility (*C*2) and (*C*3) was measured at the two mentioned compression levels.

### 2.4. Dry Anaerobic Batch Digestion Trials

Substrate material preparation—the material structure of the substrate was adjusted by intensive mixing of the dry chicken manure samples with structural material such as wood chips and wheat straw. After material conditioning the percolate digesters were filled with app. 2.0 kg (10.0–12.0 L) of the substrate mixture to a heap height of app. 0.3 m.

Dry batch anaerobic digestion—in three test series, defined dry chicken manure and structure material mixtures were fermented in two percolate digesters. Each of the individual fermentation experiments were completed after a test period of approximately four weeks. Information about the type of structure material used and the percentage of its addition to dry chicken manure can be found in Table 1. A schematic representation of the dry digestion system is depicted in Figure 2.

The testing system consisted of two percolate digesters, each connected to a percolate pump for percolate recycling. The two double walled percolate digesters each had a substrate capacity of app. 13 L and were heated to 37 °C by a thermostatically controlled water bath. The hydrolysis and methanization occurred simultaneously in the percolate digesters. Each digester was filled with 2 kg of chicken manure with and without structure material addition. The material mixture compositions that were used are shown in Table 1. The hydrolysis process was implemented by percolation of 7.6 L of percolate with a percolation rate of 660 mL/h every 30 min for approx. 20 s. In order to generate sufficient biological activity, the percolate consisted of a mixture of tap water, percolate from a pilot scale dry batch anaerobic digestion plant and percolate from lab scale dry batch digestion trials. Organic acids formed by hydrolysis were dissolved in the percolate, collected in a buffer tank and continuously irrigated on top of the substrate heap in the percolate digester. The biogas production took place in the percolate digester and buffer tank within the base of the percolate digester (shown in Figure 2). Biogas quantity was continuously measured with a gas flow meter (Ritter TG 1, Ritter GmbH Germany) and the methane content was determined with an automated gas analysis device (AWITE Gas measurement device, Germany). The volatile fatty acid concentration and the ammonia nitrogen content was regularly recorded. Specific methane yield (SMY) was calculated in accordance with VDI guideline 4630, 2006 [19] and normalized to standard conditions (dry gas, 273.15 K, 1013.25 hPa). The SMY is based on the input mass of volatile solids of the substrate mix. In the case of the control and wood chip variant, only the chicken manure VS input was considered and in case of the straw and chicken manure mixture the total VS input was used for SMY calculation. SMY originated from the volatile fatty acid content in the percolate was calculated for each percolate digester separately under the assumption that 1 g degraded volatile fatty acids in the percolate equals approx. 350 mL CH_4_. The volatile fatty acid concentration in the percolate was measured on a daily base in the first week of the test and 2 to 3 times per week after the first week.

### 2.5. Statistical Analyses

Pearson correlation analysis was performed to determine the strength of the relationship between physio-chemical characteristics (for instance material structure, percolation rate and total solids) and methane production after 35 days batch duration. Pearson’s correlation coefficients were performed using SAS software (SAS version 10.0, SAS Institute, Inc., Cary, NC, USA).

## 3. Results and Discussion

### 3.1. Material Characteristics of Chicken Manure

The umbrella term chicken manure can be differentiated and includes chicken slurry, dry chicken excreta, dried chicken excreta, chicken manure, fresh chicken excreta. The chicken manure types can differ in the origin or bird purpose and by the chicken housing style i.e., broiler, laying hens, young animals. Chicken manure is the mixture of feces and urine excreted, which contains varying amounts of undigested feeding stuff, desquamated intestinal epithelium, residues of secretion, microorganism from the intestinal flora, metabolites excreted with the urine, as well as other components e.g., feather, egg leftovers, bedding material, grid material and soil. [3]

In this study, chicken manure was tested for the material properties of total solids (TS) and volatile solids (VS), nitrogen, protein, fat and fiber composition. The 42 tested samples were derived from various sources and varied in storage age, housing form and bedding material type. The material characterization tests were carried out with the aim of obtaining an overview of the material properties and material diversity. The results are summarized in Table 2. The water content of the analyzed samples based on fresh matter (FM) averaged approximately 48.3%FM, ranging from 28.6 to 77.0%FM. Dry samples with TS contents >75% were either long term stored manure samples or collected from young animal breeding. The volatile solid content varied between approx. 56.7–86.4%, which shows the high ash content of chicken manure with approximately 30%. Sand and grit materials contained in the chicken feed and bedding material remain as ash content in the manure. Anaerobic digestion of chicken manure in continuous stirred-tank reactor (CSTR) systems can cause several technical problems. In cases of sediment accumulation, the digester volume can be reduced or technical devices such as stirrers or pumps can be negatively affected. Sediment removal can require the removal of the digester roof and the use of heavy machinery. Since dry batch anaerobic processes operate discontinuously, they are not likewise affected by sediment accumulation. After a certain retention time the digested substrate material and sediments are replaced by new fresh substrate. Moving parts such as stirrers are not present [9]. However, sediments can cause problems in percolation systems too, if the substrate permeability is reduced or fine sand migration effects the pumping and percolation system.

The Total Kjeldahl Nitrogen (TKN) content of 42 tested chicken manure samples from various sources was in a range of approx. 1.5 to 8.7%TS. On average the ammonium (NH_4_-N) content was approx. 50% of total nitrogen. The calculated raw protein content of the 42 tested samples varied between 21.6% and 46.2%TS. The material characterization results confirm the findings of Nie et.al., 2015 [8] that chicken manure is a feedstock material with an comparably high nitrogen content, which can result in process instability of the anaerobic digestion process by free ammonia inhibition The raw fat content of 39 tested samples averaged approximately 3.6% of total solids, and indicated a comparably low fat concentration while the raw fiber content of 44 tested samples ranged from approximately 14.0 to 32.5% of total solids. Several samples contained straw stalks or remains of pelletized straw or saw dust, which is commonly used as bedding material. Generally, bedding material in manure like straw or wood saw dust consists of ligno-cellulose primarily. Massé et.al. [20] confirmed the biogas potential of ligno-cellulosic biomass such as wheat straw in dry digestion processes. Hendriks and Zeeman [21] identified the crystallinity of cellulose, available surface area, and lignin content as limiting factors for the anaerobic decomposition of ligno-cellulosic biomass. In preliminary investigations [18] it was found that the fiber content can have positive effects on the feedstock permeability. The material characterization tests revealed the highly divers material composition of chicken manure and regularly feedstock testing is recommended prior anaerobic digestion.

### 3.2. Material Permeability of Chicken Manure

**Figure 3:**
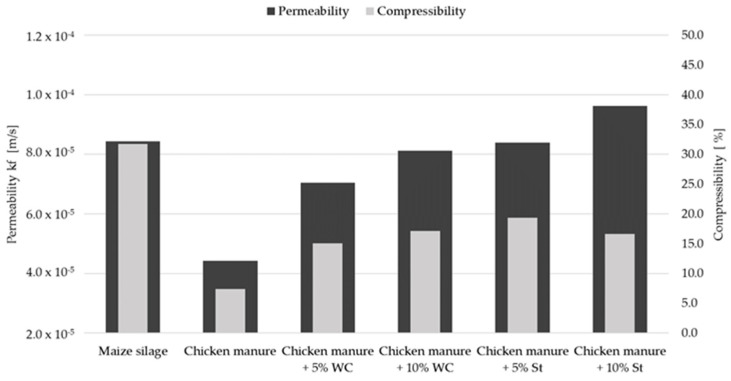
Permeability and compressibility characteristics of chicken manure with structure material addition relative to maize silage. WC, wood chips; St, straw.

The permeability and compressibility characteristics of nine chicken manure samples with and without structure material addition were determined as a measure for the feedstock suitability for percolation processes. An oedometer test apparatus was used to simulate the material compression within the substrate heap as it occurs in full scale dry batch anaerobic digestion plants. Oedometer tests without compaction (*P*1) showed similar permeability properties for all tested chicken manure samples (see Table 3). When compressive force was applied on top of the samples to simulate material compaction in lower layers of the substrate heap, the testing method revealed very different permeability and compaction properties. In order to classify and interpret the oedometer test results, the permeability of maize silage as a reference material was tested, since it is a common feedstock used successfully in several German anaerobic dry digestion biogas plants with a demonstrated sufficient permeability. Chicken manure sample No.1 was tested as impermeable under compression forces that would be caused by 1.5 m substrate heap height. Sample No.2 was tested without structure material addition and found to be impermeable under compression forces, which would be caused by 3.0 m substrate heap height. However, the addition of plastic structure carriers to sample No.2 led to a 39% superior material permeability, compared to maize silage. Sample No.3 was tested as sufficiently permeable without structure material addition. The material was collected from young animal rearing and contained high amounts of bedding material and was comparably dry at 76.5%TS. Sample No.4 without structure material addition shows a 50% lower permeability compared to maize silage. In Figure 3 it is shown that the addition of structure building materials such as wood chips and straw to chicken manure sample No.4 leads to an improvement of the percolation properties. An addition of 10% mass woodchips or 5% mass straw to chicken manure results in permeability characteristics of the feedstock comparable to maize silage.

The results shown in Table 3 indicate that in lower layers of the substrate pile, material compression increases as a result of increasing material weight and the associated pressure on top. Andre et al., 2015 [22] showed that substrate compaction leads to a reduction of the material permeability possibly due to a decrease in the material pore space. The correlation between material compression and permeability was also described by [18] for various agricultural residue materials. Some chicken manure samples from laying hens and broiler fattening were tested as impermeable when material heap height of 1–2 m was simulated. However, the results of this study confirm the findings by Hayes et al. [23] that structure materials can be used to increase the permeability of the feedstock mixture and thus improve the suitability of substrates with low material permeability for percolation processes. The correlations between physio-chemical parameters and permeability revealed that single physio-chemical characteristics did not significantly correlate with material permeability, indicating that the material permeability could be dependent on the storage age, housing form and bedding material type. The detailed results of Pearson’s correlation coefficients are presented in supplementary Table S1. Chicken manure with a high proportion of dry fibrous bedding material could have a better material suitability for percolation processes compared to the sample materials, which were composed mainly of chicken manure alone.

The aim of this preliminary investigation was to determine the suitability of the material characteristics in terms of permeability under compacting forces and compactibility of chicken manure used in dry batch anaerobic digestion processes. In practice, there is a risk of technical problems if percolate cannot pass through the substrate material in the percolate digester. As a result, dead zones can occur in the lower part of the substrate material bed. In the worst case scenarios, large areas of the substrate heap are not sufficiently percolated, which can lead to deteriorated organic degradation or to the accumulation of percolate within the percolate digester [24]. However, further tests at full scale are required to determine if the oedometer testing apparatus results can be transferred completely to full scale. Further digestion trials should be conducted in order to determine if structure stability of the input material sustains during the digestion process and if the digestate properties can be manipulated by a targeted adjustment of the substrate properties by structure material addition. Positive results would be of interest for the handling and transportation of fermentation residues, since a very moist and structurally weak digestate may require more time for dewatering [18]. Improved dewaterability of structurally optimized chicken manure mixtures could contribute to an increased plant throughput in anaerobic dry digestion processes, since fermentation residues can only be removed from the percolator at the end of fermentation after the contained percolate has run off. Permeability and dewaterability tests of digestate samples were not part of the study, but preliminary tests at lab scale showed that structure material addition to the input material resulted in better percolate drainage [18].

### 3.3. Results of Dry Batch Anaerobic Digestion Trials

The aim of the digestion trials was to determine the biogas generation of chicken manure in a one step dry bath anaerobic digestion process without inoculum addition and a secondary methanization digester. A further aim was to determine the influence of structure material addition on the substrate methane yield. For this purpose, six dry batch anaerobic digestion trials were carried out with substrate mixtures of dry chicken manure and different additions of the structure materials straw and wood chips.

#### 3.3.1. Process Conditions

**Figure 4:**
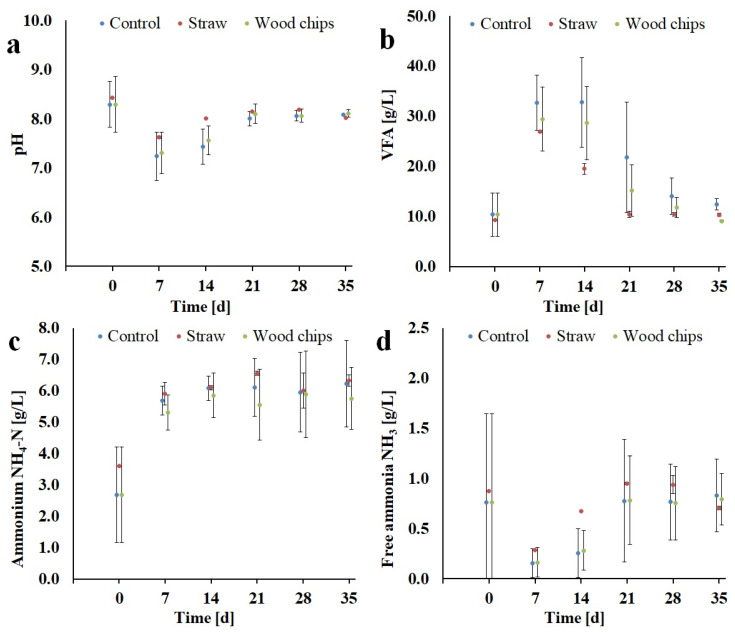
**a–d.** Process conditions during dry batch anaerobic digestion of chicken manure. pH value (**a**), volatile fatty acid concentration (**b**), ammonium concentration (**c**), free ammonia concentration (**d**).

The pH varied between 6.8 to 8.2 during the course of the digestion experiments (Figure 4a). During the first two weeks of the trial the pH-value decreased and reached the lowest values after approximately 7 days. During the second and third week the pH value increased and steadied until the end of the test. The graphs indicate that the pH-value was affected by the volatile fatty acid (VFA) and ammonium nitrogen concentration. Volatile fatty acid concentrations in the percolate increased during the test duration to a maximum of 32.8 g/L but decreased to the starting value of approx. 10 g/L in less than 30 days (Figure 4b). This could indicate that a secondary methanation step was not required to reduce the volatile components temporarily accumulated in the percolate. The buffer capacity of the percolate was sufficient to stabilise the pH-value above 6.8 even at peak VFA concentrations. The ammonium nitrogen (NH_4_-N) concentrations ranged from 1.6 to a maximum of 7.2 g/L (Figure 4c) and free ammonia (FA) ranged from 0.1 to 1.2 g/L (Figure 4d). According to Hafner and Bisogni [25], free ammonia calculation for liquid digestate or percolate using the described formula can lead in some cases to an overestimation of the FA, as percolate has a rather complex material composition compared to water. Ammonium (NH_4_+) and free ammonia (NH_3_) are the predominant forms of inorganic nitrogen. Ammonium nitrogen (NH_4_-N) is a degradation product of organic nitrogen such as proteins or urea during anaerobic digestion. In the digester liquid, NH_4_-N is present as ammonium ions (NH_4_+) and as free ammonia (NH3). Increasing pH or temperature, results in a higher percentage of NH_4_-N present as free ammonia (NH_3_). It has been shown that free ammonia is more toxic, as it can pass through the cell membrane, causing proton imbalance and potassium deficiency [26]. According to Ying et.al [26], Bujoczek et.al. [27] and Yenigün and Demirel [28], several research groups report that inhibitory NH_4_-N concentration range between 1.5 to 5.0 g/L. However, significantly higher concentrations of up to 14 g/L are possible by microbial adaptation of the digestion process. Niu et al. [29] described a successfully recovery from ammonia inhibition of a CSTR system operated with chicken manure as substrate at TAN concentrations of 16 g/L after dilution and different washing steps. In the case of our study, an inhibition of the process biology can be expected, which possibly negatively effected the methane production.

#### 3.3.2. Specific Methane Yield

In our study, chicken manure achieved an average SMY of 135 ± 20 mL/g VS or 75 ± 5 mL/g FM. According to FNR [6], the average biochemical methane potential of chicken manure is 280 mL/g VS or 90 mL/g FM. Several researchers investigated the SMY of chicken manure with or without bedding material in dry batch anaerobic processes. The SMY of chicken manure measured by Marchioro et.al. [30] was 74 mL/g VS and lower compared to the findings of this study. Other authors such as Bi et al. [31] or Wang et al. [32] published considerable higher methane yields of 280 mL/g VS and 437 mL/g VS respectively, partly due to ammonia extraction within the process. One possible reason for divergent results in various studies is the differences in physical and chemical composition of the manure, due to the method of rearing as well as the utilisation and kind of bedding material.

The methane concentration increased during the test duration to a maximum of approx. 68% as shown in Table S3.

**Figure 5:**
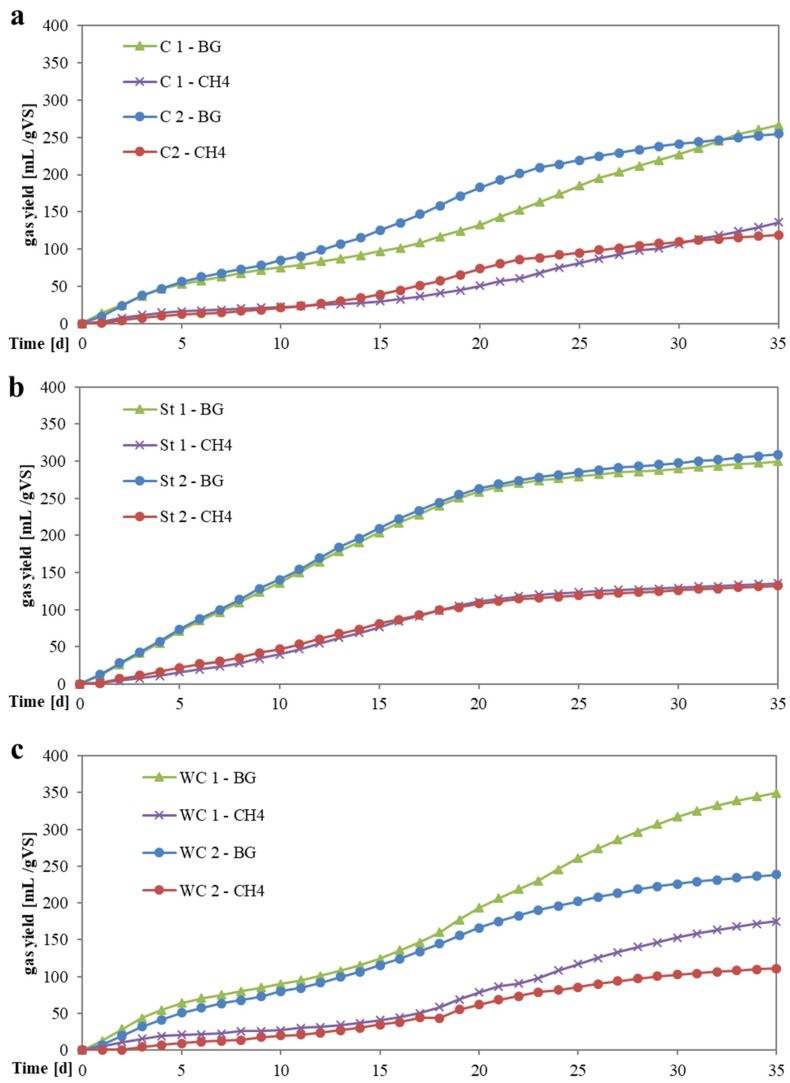
(**a**–**c**) Specific yield of biogas (BG) and methane (CH4) of chicken manure in dry batch anaerobic digestion. C, control variant (**a**); St, straw variant (**b**); WC, wood chip variant (**c**).

In our study, chicken manure without structure material addition achieved SMYs of 127 ± 12 mL/g VS or 70 ± 6 mL/g FM (Table 4). The Figure 5a–c shows the cumulated daily biogas and methane production of the percolate digester and the calculated SMY of the VFA contained in the percolate. As depicted in Figure 5a, the cumulative methane production showed a low methane production during the first two weeks. Thereafter the methane production increased but the biogas production rate was not identical in the two parallel trials. The degradation of the organic content was possibly different due to an uneven percolate flow or distribution within the substrate material. However, the measured specific methane yield of the parallel trial was in a comparable range after 35 days. The biogas production did not reach a plateau phase at the end of the test. A longer test duration would have led to a higher methane production. However, retention times in dry batch processes in practice are commonly shorter. The digestion trials showed that dry batch anaerobic chicken manure digestion was possible, even without inoculum addition. However, an addition of inoculum to substrate material in the beginning could help to accelerate the initial methane production rate.

The SMY of the straw variants was 6% higher compared to the control variant without structure material addition and resulted in 135 ± 4 mL/g VS or 75 ± 2 mL/g FM. Methane production occurred without a lag phase in the beginning. The methane concentration of the biogas exceeded 50% after a test duration of approx. 8 days. Data to the gas composition is summarized in Table S3. The control variant without structure material addition and the chicken manure mixture with wood chip addition showed a lower methane production rate and a methane concentration of 50%was reached after approx. 16 days test duration. As depicted in Figure 5b, a two-step gas production was observed in the straw variants with a first phase between the test start and day 20, and a second phase with a lower but steady methane production between day 20 and day 35. A possible reason could be the organic degradation of the additional straw content. Even though the added content of straw was different in both digesters (5% and 10% of the mass), the gas production was similar during the course of the trial. Possibly the straw addition to the chicken manure lead also to an increase of the pore space within the substrate mixture resulting in an even percolate distribution and yielded a comparable degradation of the organic content.

The digestion of the wood chip variants resulted in the highest SMY with 143 ± 45 mL/g VS or 79 ± 25 mL/g FM. Although the average methane generation was 11% higher compared to the control without structure material addition, the high standard deviation between the two digesters has to be considered. Until day 20, the methane production rate was similar to the control variant. Between day 21 and 35, the methane production increased but varied in the parallel digesters (see Figure 5c). The preliminary tests showed that wood chip addition to the chicken manure of 10% mass led to a very coarse substrate mixture. The material mixture possibly promotes channel effects, which results in percolate passing the substrate mix without an even distribution in the substrate heap, leading to only a partial organic degradation. Channel effects were observed in the material characterization tests when proportions of 10% mass wood chips where added to the substrate. Pearson’s correlation showed a positive, but weak (r2 = 0.54), correlation between material permeability under compression (P3) and specific methane yield. Thus, chicken manure mixtures with wood chip addition showed best material permeability with 8.02 × 10^−5^ m/s and achieved the highest specific methane yield. The detailed results of results of Pearson’s correlation coefficients are presented in supplementary Table S2.

It must be checked whether a very high use of structural substances is possible in full scale plant operation, because this reduces the plant throughput of the actual feedstock. Only very cost-effective structural materials, such as residual straw, were considered, since alternative structural materials, such as wood chips, are highly unlikely to be economically viable in practical application. A recovery of structural materials directly from the digestate is possible but challenging due to the unfavorable material properties that will impact the subsequent sieving. Under practical conditions, the use of screened residues as structure building material from chicken manure with bedding material appears to be more promising. In order to replace structural materials, which do not contribute to gas production in the mixture the material permeability of chicken manure can also be improved by mixing fibrous bedding material containing chicken manure with a high proportion of lignified biomass with moist, structurally weak chicken manure without bedding material, if both types of chicken manure are available simultaneously. A validation of the findings in full scale operations should be considered.

## 4. Conclusions

Material characterization tests of chicken manure were carried out to determine the feedstock suitability in terms of feedstock composition, permeability under compaction and compressibility for dry batch anaerobic digestion processes. Oedometer tests under compression showed that the permeability and structural stability of chicken manure can be improved through an initial addition of structural materials such as wood chips or wheat straw to the substrate mixture. Even originally impermeable chicken manure samples were suitable for percolation processes after an addition of 5% straw or wood chips (by mass). Channel effects with uneven percolate distribution were observed on a number of samples with 10% woodchips addition. Anaerobic dry batch digestion trials were carried out to investigate the biogas generation of dry chicken manure without inoculation and separate methanization step. The conducted digestion trials showed that dry batch anaerobic chicken manure digestion was possible without inoculum addition. However, the methane production showed a lag phase of approximately 2 weeks after the test start, and the measured methane yields were lower compared to published SMYs for wet digestion systems. In this study the obtained methane yield of chicken manure averaged 135 ± 20 mL/g VS or 75 ± 5 mL/g FM, which represents 70% of the gas potential based on fresh mass of common energy crops such as corn silage. The addition of straw and wood chips led to an increase of the specific methane yield by 6% and 11%, respectively.

## Figures and Tables

**Table 1:** Dry batch anaerobic digestion trials and structure material addition.

	Designation	Structure Material	Structure Material Addition (w%)
Trial 1	*C*1	-	-
Trial 2	*C*2	-	-
Trial 3	*S*1	Straw	5
Trial 4	*S*2	Straw	10
Trial 5	*W*1	Woodchips	10
Trial 6	*W*2	Woodchips	10

**Table 2:** Material characteristics of chicken manure.

	TS	VS	NH_4_-N	TKN	Raw Protein	Raw Fat	Raw Fibre
	*n* = 50	*n* = 50	*n* = 44	*n* = 42	*n* = 42	*n* = 38	*n* = 44
	%FM	%TS	%TS	%TS	%TS	%TS	%TS
Mean	48.3	69.5	0.8	4.7	21.6	3.6	14.0
Max	77.0	86.4	2.3	8.7	46.2	12.9	32.5
Min	28.6	56.7	0.1	1.5	1.5	0.1	1.7

*n* number of tested chicken manure samples obtained from various sources.

**Table 3:** Permeability of chicken manure with and without structure material addition.

Sample	Structure Material	*P*1	*P*2	*C*2	*P*3	*C*3
Material	Addition [w%]	[m/s]	[m/s]	[%]	[m/s]	[%]
MS	-	1.59 × 10^−4^	1.08 × 10^−4^	22.94	8.39 × 10^−5^	31.76
1	-	1.91 × 10^−4^	*n.p.*	*n*.m.	*n.p.*	*n*.m.
2	-	4.36 × 10^−5^	9.58 × 10^−6^	*n*.m.	*n.p.*	*n*.m.
2	5 (PSC)	2.14 × 10^−4^	1.36 × 10^−4^	4.47	1.38 × 10^−4^	19.42
3	-	1.91 × 10^−4^	1.38 × 10^−4^	8.43	9.89 × 10^−5^	18.54
4	-	1.02 × 10^−4^	6.34 × 10^−5^	3.28	4.32 × 10^−5^	7.38
4	5 (WC)	1.43 × 10^−4^	9.95 × 10^−5^	7.53	6.87 × 10^−5^	15.07
4	10 (WC)	1.49 × 10^−4^	1.01 × 10^−4^	13.70	8.02 × 10^−5^	17.12
4	5 (St)	1.71 × 10^−4^	1.10 × 10^−4^	15.00	8.20 × 10^−5^	19.38
4	10 (St)	1.80 × 10^−4^	1.15 × 10^−4^	14.29	9.39 × 10^−5^	16.67

*p* permeability and *C* compressibility (without compaction (*P*1), 1.5 and 3.0 m simulated material height (*P*2 and *C*2, *P*3 and *C*3); MS Maize silage; PSC Plastic structure carriers; WC wood chips; St straw; Sample 1-from Laying hens; 2-Broiler fattening; 3-Young animals; 4-Laying hens.

**Table 4:** Specific methane yield (SMY) of chicken manure with and without additives measured in dry batch anerobic digestion trials.

	SMY_1_	SMY_2_	SMY_mean_	
	[mL/g VS]	[mL/g VS]	[mL/g VS]	[mL/g FM]
Control	136	119	127 ± 12	70 ± 6
Straw	138	132	135 ± 4	75 ± 2
Woodchips	175	111	143 ± 45	79 ± 25

## Supplementary Materials

The following are available online at https://www.mdpi.com/2306-5354/7/3/106/s1, Table S1: Pearson’s correlation coefficients of the physio-chemical charactieristics of chicken manure with material permeability, Table S2: Pearson’s correlation coefficients of material permeability and compressibility with specific methane yield. Table S3: Biogas and methane production and gas composition the dry digestion of chicken manure with and without structure material addition.
