# Supplementary material for: Material Characterization and Substrate Suitability Assessment of Chicken Manure for Dry Batch Anaerobic Digestion Processes
Source: Bioengineering (Basel). 2020 Sep 7;7(3):106. doi: 10.3390/bioengineering7030106 (PMC7552755; doi:10.3390/bioengineering7030106)
Supplement: Supplementary file 1 [file bioengineering-07-00106-s001.zip › Table S2.docx]

|  | **P1** | **P2** | **C2** | **P3** | **C3** |
| --- | --- | --- | --- | --- | --- |
| **P2** | 0.98** |  | 0.90* | 0.97** | 0.94* |
| **C2** | 0.92* | 0.90* |  | 0.94* | 0.93* |
| **P3** | 0.98** | 0.97** | 0.94* |  | 0.90* |
| **C3** | 0.91* | 0.94* | 0.93* | 0.90* |  |
| **SMY** | 0.47 | 0.56 | 0.72 | 0.55 | 0.76 |

**Table S2.** Pearson’s correlation coefficients of material permeability and compressibility with specific methane yield. Statistically significant values are indicated by symbols: **P < 0.01; *P < 0.05. P, permeability and C, compressibility (without compaction (P1), 1.5 and 3.0 m simulated material height (P2 and C2, P3 and C3); SMY, specific methane yield.
