# Supplementary material for: Material Characterization and Substrate Suitability Assessment of Chicken Manure for Dry Batch Anaerobic Digestion Processes
Source: Bioengineering (Basel). 2020 Sep 7;7(3):106. doi: 10.3390/bioengineering7030106 (PMC7552755; doi:10.3390/bioengineering7030106)
Supplement: Supplementary file 1 [file bioengineering-07-00106-s001.zip › Table S3.docx]

|  |  |  |  |  | Gas composition | | | | |
| --- | --- | --- | --- | --- | --- | --- | --- | --- | --- |
| C1 | Time | Biogas | Methane | Methane + VFA | CH4 | O2 | CO2 | H2 | H2S |
|  | d | mL/g VS | mL/g VS | mL/g VS | % | % | % | ppm | ppm |
|  | 0 | 0 | 0 | 0 |  |  |  |  |  |
|  | 1 | 14 | 1 | 3 | 26 | 4 | 66 | 31461 | 2684 |
|  | 2 | 25 | 3 | 8 | 12 | 21 | 63 | 28377 | 4384 |
|  | 3 | 37 | 5 | 12 | 15 | 25 | 58 | 7933 | 18919 |
|  | 4 | 47 | 6 | 15 | 19 | 11 | 67 | 2315 | 20615 |
|  | 5 | 53 | 7 | 17 |  |  |  |  |  |
|  | 6 | 58 | 8 | 18 |  |  |  |  |  |
|  | 7 | 63 | 9 | 19 |  |  |  |  |  |
|  | 8 | 68 | 10 | 20 |  |  |  |  |  |
|  | 9 | 72 | 11 | 21 | 24 | 1 | 73 | 0 | 22866 |
|  | 10 | 75 | 12 | 22 |  |  |  |  |  |
|  | 11 | 79 | 13 | 24 | 28 | 0 | 69 | 0 | 20752 |
|  | 12 | 83 | 14 | 25 |  |  |  |  |  |
|  | 13 | 87 | 16 | 26 |  |  |  |  |  |
|  | 14 | 92 | 17 | 28 |  |  |  |  |  |
|  | 15 | 97 | 19 | 30 |  |  |  |  |  |
|  | 16 | 102 | 22 | 33 | 46 | 0 | 52 | 0 | 13852 |
|  | 17 | 108 | 26 | 37 |  |  |  |  |  |
|  | 18 | 117 | 30 | 41 | 57 | 0 | 42 | 0 | 9309 |
|  | 19 | 125 | 34 | 45 |  |  |  |  |  |
|  | 20 | 132 | 40 | 51 |  |  |  |  |  |
|  | 21 | 143 | 46 | 57 |  |  |  |  |  |
|  | 22 | 153 | 53 | 61 | 65 | 0 | 34 | 0 | 3489 |
|  | 23 | 163 | 60 | 68 | 66 | 0 | 33 | 0 | 3125 |
|  | 24 | 174 | 67 | 75 | 66 | 0 | 34 | 0 | 1685 |
|  | 25 | 185 | 74 | 82 | 65 | 0 | 35 | 0 | 1113 |
|  | 26 | 195 | 80 | 87 |  |  |  |  |  |
|  | 27 | 203 | 86 | 93 |  |  |  |  |  |
|  | 28 | 212 | 91 | 98 |  |  |  |  |  |
|  | 29 | 220 | 96 | 101 | 68 | 0 | 31 | 0 | 2537 |
|  | 30 | 227 | 102 | 107 |  |  |  |  |  |
|  | 31 | 236 | 109 | 113 | 69 | 0 | 30 | 0 | 2635 |
|  | 32 | 245 | 115 | 119 | 69 | 0 | 30 | 0 | 2635 |
|  | 33 | 254 | 120 | 124 |  |  |  |  |  |
|  | 34 | 261 | 126 | 130 |  |  |  |  |  |
|  | 35 | 267 | 132 | 136 | 68 | 1 | 31 | 0 |  |
|  |  |  |  |  |  |  |  |  |  |
| C2 | 0 | 0 | 0 | 0 |  |  |  |  |  |
|  | 1 | 10 | 0 | 0 | 5 | 1 | 91 | 25341 |  |
|  | 2 | 24 | 1 | 4 | 4 | 1 | 96 |  |  |
|  | 3 | 37 | 2 | 8 | 11 | 1 | 86 | 14447 | 7257 |
|  | 4 | 47 | 3 | 10 | 10 | 0 | 88 | 2557 | 18052 |
|  | 5 | 56 | 5 | 12 | 14 | 1 | 85 |  |  |
|  | 6 | 63 | 6 | 13 | 18 | 1 | 81 |  |  |
|  | 7 | 68 | 7 | 14 | 22 | 0 | 78 | 0 |  |
|  | 8 | 73 | 8 | 17 | 22 | 0 | 76 | 491 | 17802 |
|  | 9 | 78 | 10 | 18 | 30 | 1 | 69 |  |  |
|  | 10 | 85 | 12 | 21 | 34 | 1 | 65 |  |  |
|  | 11 | 91 | 14 | 23 | 38 | 1 | 61 |  |  |
|  | 12 | 99 | 18 | 27 | 43 | 1 | 57 |  |  |
|  | 13 | 107 | 21 | 31 |  |  |  |  |  |
|  | 14 | 115 | 25 | 34 | 48 | 0 | 51 |  |  |
|  | 15 | 126 | 31 | 39 | 51 | 0 | 48 | 196 |  |
|  | 16 | 136 | 36 | 45 | 55 | 0 | 44 | 182 |  |
|  | 17 | 147 | 43 | 51 | 59 | 0 | 40 | 171 |  |
|  | 18 | 158 | 50 | 57 | 62 | 0 | 37 | 167 |  |
|  | 19 | 171 | 59 | 66 | 66 | 0 | 33 |  |  |
|  | 20 | 183 | 66 | 73 | 69 | 0 | 31 |  |  |
|  | 21 | 193 | 73 | 80 |  |  |  |  |  |
|  | 22 | 201 | 79 | 86 | 69 | 0 | 30 | 137 |  |
|  | 23 | 209 | 85 | 89 | 70 | 0 | 30 | 136 |  |
|  | 24 | 214 | 88 | 92 | 70 | 0 | 30 | 126 |  |
|  | 25 | 219 | 92 | 95 | 72 | 2 | 25 | 116 |  |
|  | 26 | 225 | 96 | 99 | 70 | 1 | 29 | 115 |  |
|  | 27 | 229 | 99 | 102 | 69 | 1 | 30 | 110 |  |
|  | 28 | 234 | 102 | 105 | 69 | 1 | 31 | 100 |  |
|  | 29 | 238 | 105 | 108 | 68 | 1 | 31 | 93 |  |
|  | 30 | 241 | 107 | 110 | 68 | 1 | 30 | 87 |  |
|  | 31 | 244 | 109 | 112 |  |  |  |  |  |
|  | 32 | 247 | 111 | 113 | 69 | 1 | 30 | 77 |  |
|  | 33 | 249 | 113 | 115 |  |  |  |  |  |
|  | 34 | 252 | 114 | 117 | 68 | 1 | 31 | 72 |  |
|  | 35 | 255 | 116 | 119 |  |  |  |  |  |
|  |  |  |  |  |  |  |  |  |  |
| St1 | 0 | 0 | 0 | 0 | 0 | 0 | 0 | 0 | 0 |
|  | 1 | 11 | 2 | 5 | 19 | 6 | 74 | 10132 | 284 |
|  | 2 | 26 | 4 | 8 | 19 | 1 | 81 | 1854 | 812 |
|  | 3 | 41 | 8 | 13 | 23 | 0 | 77 | 1344 | 2676 |
|  | 4 | 55 | 11 | 17 | 26 | 0 | 73 | 0 | 5445 |
|  | 5 | 72 | 16 | 23 |  |  |  |  |  |
|  | 6 | 85 | 20 | 27 |  |  |  |  |  |
|  | 7 | 97 | 24 | 31 |  |  |  |  |  |
|  | 8 | 110 | 28 | 37 | 40 | 1 | 58 | 0 | 10689 |
|  | 9 | 124 | 34 | 42 | 45 | 0 | 54 | 0 | 10560 |
|  | 10 | 136 | 40 | 48 | 49 | 1 | 49 | 0 | 10312 |
|  | 11 | 150 | 47 | 54 | 53 | 0 | 46 | 0 | 9196 |
|  | 12 | 164 | 54 | 62 |  |  |  |  |  |
|  | 13 | 179 | 62 | 70 |  |  |  |  |  |
|  | 14 | 191 | 69 | 76 |  |  |  |  |  |
|  | 15 | 204 | 76 | 83 | 64 | 0 | 35 | 0 | 7225 |
|  | 16 | 217 | 84 | 91 | 67 | 0 | 32 | 0 | 6118 |
|  | 17 | 228 | 91 | 98 | 68 | 0 | 31 | 0 | 6312 |
|  | 18 | 240 | 99 | 103 | 68 | 1 | 31 | 0 | 5700 |
|  | 19 | 251 | 105 | 110 |  |  |  |  |  |
|  | 20 | 259 | 111 | 115 |  |  |  |  |  |
|  | 21 | 265 | 114 | 119 |  |  |  |  |  |
|  | 22 | 270 | 118 | 121 |  |  |  |  |  |
|  | 23 | 274 | 120 | 123 | 68 | 0 | 31 | 0 | 4519 |
|  | 24 | 277 | 122 | 125 | 66 | 1 | 33 | 0 |  |
|  | 25 | 279 | 123 | 126 |  |  |  |  |  |
|  | 26 | 282 | 125 | 128 |  |  |  |  |  |
|  | 27 | 285 | 126 | 129 |  |  |  |  |  |
|  | 28 | 286 | 127 | 130 | 65 | 1 | 34 | 0 |  |
|  | 29 | 288 | 128 | 131 | 65 | 1 | 34 | 0 |  |
|  | 30 | 290 | 130 | 133 | 67 | 1 | 31 | 0 | 3617 |
|  | 31 | 292 | 131 | 134 | 64 | 1 | 35 | 0 |  |
|  | 32 | 294 | 132 | 135 | 63 | 2 | 35 | 0 |  |
|  | 33 | 296 | 133 | 136 |  |  |  |  |  |
|  | 34 | 298 | 134 | 137 |  |  |  |  |  |
|  | 35 | 298 | 135 | 138 | 63 | 1 | 36 | 0 |  |
|  |  |  |  |  |  |  |  |  |  |
| St2 | 0 | 0 | 0 | 0 | 0 | 0 | 0 | 0 | 0 |
|  | 1 | 13 | 1 | 1 | 14 | 7 | 78 | 99 | 406 |
|  | 2 | 28 | 4 | 7 | 19 | 1 | 80 | 95 | 869 |
|  | 3 | 43 | 7 | 11 | 23 | 1 | 76 | 0 | 2443 |
|  | 4 | 57 | 10 | 16 | 26 | 0 | 73 | 0 | 4751 |
|  | 5 | 74 | 15 | 22 |  |  |  |  |  |
|  | 6 | 88 | 19 | 27 |  |  |  |  |  |
|  | 7 | 100 | 22 | 30 |  |  |  |  |  |
|  | 8 | 114 | 27 | 35 | 41 | 1 | 57 | 0 | 9932 |
|  | 9 | 128 | 32 | 42 | 46 | 0 | 53 | 0 | 9912 |
|  | 10 | 141 | 38 | 47 | 51 | 0 | 48 | 0 | 9751 |
|  | 11 | 154 | 44 | 53 | 55 | 0 | 44 | 0 | 9222 |
|  | 12 | 169 | 52 | 60 |  |  |  |  |  |
|  | 13 | 184 | 59 | 68 |  |  |  |  |  |
|  | 14 | 196 | 65 | 74 |  |  |  |  |  |
|  | 15 | 209 | 72 | 81 | 65 | 0 | 34 | 0 | 6158 |
|  | 16 | 223 | 80 | 86 | 67 | 0 | 32 | 0 | 5928 |
|  | 17 | 233 | 86 | 92 | 68 | 0 | 31 | 0 | 5292 |
|  | 18 | 245 | 93 | 99 | 68 | 0 | 31 | 0 | 4862 |
|  | 19 | 255 | 98 | 103 |  |  |  |  |  |
|  | 20 | 263 | 103 | 108 |  |  |  |  |  |
|  | 21 | 269 | 107 | 111 |  |  |  |  |  |
|  | 22 | 274 | 110 | 114 |  |  |  |  |  |
|  | 23 | 279 | 112 | 116 | 68 | 1 | 31 | 0 | 3936 |
|  | 24 | 282 | 114 | 117 | 65 | 2 | 32 | 0 |  |
|  | 25 | 285 | 116 | 119 |  |  |  |  |  |
|  | 26 | 288 | 117 | 121 |  |  |  |  |  |
|  | 27 | 291 | 119 | 123 |  |  |  |  |  |
|  | 28 | 293 | 120 | 123 | 65 | 1 | 33 | 0 |  |
|  | 29 | 295 | 121 | 125 | 65 | 1 | 34 | 0 |  |
|  | 30 | 298 | 123 | 126 | 67 | 0 | 32 | 0 | 3297 |
|  | 31 | 300 | 124 | 128 | 64 | 1 | 35 | 0 |  |
|  | 32 | 302 | 125 | 128 | 64 | 1 | 35 | 0 |  |
|  | 33 | 305 | 126 | 130 |  |  |  |  |  |
|  | 34 | 307 | 127 | 131 |  |  |  |  |  |
|  | 35 | 309 | 129 | 132 | 64 | 1 | 35 | 0 |  |
|  |  |  |  |  |  |  |  |  |  |
| WC1 | 0 | 0 | 0 | 0 | 0 | 0 | 0 | 0 | 0 |
|  | 1 | 12 | 3 | 6 | 26 | 4 | 66 | 31461 | 2684 |
|  | 2 | 29 | 5 | 11 | 12 | 21 | 63 | 28377 | 4384 |
|  | 3 | 43 | 7 | 15 | 15 | 25 | 58 | 7933 | 18919 |
|  | 4 | 55 | 10 | 19 | 19 | 11 | 67 | 2315 | 20615 |
|  | 5 | 64 | 11 | 21 |  |  |  |  |  |
|  | 6 | 70 | 13 | 22 |  |  |  |  |  |
|  | 7 | 75 | 14 | 23 |  |  |  |  |  |
|  | 8 | 80 | 15 | 26 |  |  |  |  |  |
|  | 9 | 85 | 16 | 26 | 24 | 1 | 73 | 0 | 22866 |
|  | 10 | 90 | 17 | 27 |  |  |  |  |  |
|  | 11 | 95 | 19 | 30 | 28 | 0 | 69 | 0 | 20752 |
|  | 12 | 101 | 21 | 31 |  |  |  |  |  |
|  | 13 | 108 | 24 | 34 |  |  |  |  |  |
|  | 14 | 115 | 26 | 37 |  |  |  |  |  |
|  | 15 | 124 | 30 | 41 |  |  |  |  |  |
|  | 16 | 136 | 35 | 45 | 46 | 0 | 52 | 0 | 13852 |
|  | 17 | 146 | 40 | 50 |  |  |  |  |  |
|  | 18 | 160 | 48 | 58 | 57 | 0 | 42 | 0 | 9309 |
|  | 19 | 177 | 59 | 69 |  |  |  |  |  |
|  | 20 | 193 | 68 | 78 |  |  |  |  |  |
|  | 21 | 206 | 77 | 87 |  |  |  |  |  |
|  | 22 | 219 | 85 | 91 | 65 | 0 | 34 | 0 | 3489 |
|  | 23 | 230 | 92 | 98 | 66 | 0 | 33 | 0 | 3125 |
|  | 24 | 246 | 103 | 108 | 66 | 0 | 34 | 0 | 1685 |
|  | 25 | 261 | 112 | 117 | 65 | 0 | 35 | 0 | 1113 |
|  | 26 | 274 | 121 | 125 |  |  |  |  |  |
|  | 27 | 286 | 129 | 133 |  |  |  |  |  |
|  | 28 | 297 | 136 | 140 |  |  |  |  |  |
|  | 29 | 307 | 143 | 147 | 68 | 0 | 31 | 0 | 2537 |
|  | 30 | 317 | 150 | 153 |  |  |  |  |  |
|  | 31 | 325 | 155 | 159 | 69 | 0 | 30 | 0 | 2635 |
|  | 32 | 332 | 161 | 163 | 69 | 0 | 30 | 0 | 2635 |
|  | 33 | 339 | 165 | 168 |  |  |  |  |  |
|  | 34 | 345 | 169 | 172 |  |  |  |  |  |
|  | 35 | 350 | 173 | 175 | 68 | 1 | 31 | 0 |  |
|  |  |  |  |  |  |  |  |  |  |
| WC2 | 0 | 0 | 0 | 0 | 0 | 0 | 0 | 0 | 0 |
|  | 1 | 7 | 0 | 0 | 3 | 3 | 90 | 100 |  |
|  | 2 | 20 | 1 | 1 | 3 | 1 | 97 |  |  |
|  | 3 | 32 | 1 | 1 | 7 | 1 | 89 | 100 | 6067 |
|  | 4 | 41 | 2 | 2 | 9 | 0 | 89 | 96 | 12250 |
|  | 5 | 51 | 4 | 4 | 14 | 1 | 85 |  |  |
|  | 6 | 57 | 5 | 5 | 17 | 0 | 83 |  |  |
|  | 7 | 63 | 6 | 6 | 21 | 0 | 79 | 0 |  |
|  | 8 | 68 | 7 | 7 | 21 | 0 | 77 |  | 14258 |
|  | 9 | 73 | 8 | 8 | 30 | 1 | 69 |  |  |
|  | 10 | 80 | 11 | 11 | 33 | 0 | 66 |  |  |
|  | 11 | 84 | 12 | 12 | 37 | 0 | 63 |  |  |
|  | 12 | 92 | 15 | 15 | 41 | 0 | 58 |  |  |
|  | 13 | 99 | 19 | 19 |  |  |  |  |  |
|  | 14 | 106 | 22 | 22 | 47 | 0 | 53 |  |  |
|  | 15 | 115 | 26 | 26 | 50 | 0 | 49 | 60 | 7635 |
|  | 16 | 124 | 31 | 31 | 55 | 0 | 44 | 55 | 6364 |
|  | 17 | 134 | 37 | 37 | 59 | 0 | 40 | 53 | 6168 |
|  | 18 | 144 | 44 | 44 | 62 | 0 | 37 | 53 | 5302 |
|  | 19 | 156 | 51 | 51 | 66 | 0 | 34 |  |  |
|  | 20 | 166 | 58 | 58 | 68 | 0 | 32 |  |  |
|  | 21 | 175 | 64 | 64 | 69 | 0 | 30 |  |  |
|  | 22 | 183 | 69 | 69 | 68 | 1 | 31 | 47 | 3387 |
|  | 23 | 191 | 75 | 75 | 69 | 1 | 30 | 47 | 3145 |
|  | 24 | 196 | 79 | 79 | 68 | 1 | 30 | 45 | 3049 |
|  | 25 | 202 | 83 | 83 | 71 | 1 | 28 | 46 | 4 |
|  | 26 | 208 | 87 | 87 |  |  |  |  |  |
|  | 27 | 213 | 91 | 91 |  |  |  |  |  |
|  | 28 | 218 | 94 | 94 |  |  |  |  |  |
|  | 29 | 223 | 97 | 97 | 70 | 1 | 29 | 40 |  |
|  | 30 | 226 | 100 | 100 |  |  |  |  |  |
|  | 31 | 229 | 102 | 102 |  |  |  |  |  |
|  | 32 | 232 | 104 | 104 |  |  |  |  |  |
|  | 33 | 234 | 105 | 105 |  |  |  |  |  |
|  | 34 | 236 | 107 | 107 |  |  |  |  |  |
|  | 35 | 238 | 108 | 108 | 66 | 1 | 32 | 35 | 2434 |

**Table S3.** Biogas and methane production and gas composition the dry digestion of chicken manure with and without structure material addition. Methane + VFA, measured specific methane yield (SMY) of the percolate digester and calculated SMY of the VFA contained in the percolate; C 1 and 2, control without structure material addition; WC, wood chip addition, St, straw addition
