# Supplementary material for: Material Characterization and Substrate Suitability Assessment of Chicken Manure for Dry Batch Anaerobic Digestion Processes
Source: Bioengineering (Basel). 2020 Sep 7;7(3):106. doi: 10.3390/bioengineering7030106 (PMC7552755; doi:10.3390/bioengineering7030106)
Supplement: Supplementary file 1 [file bioengineering-07-00106-s001.zip › Table S1.docx]

|  | **NFC** | **Cell** | **Lign** | **Hemi** | **NDF** | **ADF** | **RA** | **RP** | **RF** | **Rfi** | **NfE** | **TS** | **VS** | **N** | **TKN** | **BD** | **P1** | **P4** |
| --- | --- | --- | --- | --- | --- | --- | --- | --- | --- | --- | --- | --- | --- | --- | --- | --- | --- | --- |
| **NFC** |  | 0.60 | 0.01 | -0.49 | 0.36 | 0.42 | -0.69 | -0.37 | 0.21 | -0.08 | 1.00^**^ | 0.39 | 0.69 | -0.80 | -0.49 | 0.01 | -0.10 | 0.99^*^ |
| **Cel** | 0.60 |  | 0.72 | -0.93 | 0.96^*^ | 0.96^*^ | -0.99^**^ | 0.52 | 0.90 | 0.60 | 0.55 | -0.43 | 0.99^**^ | -0.03 | 0.40 | 0.56 | 0.00 | 0.66 |
| **Lig** | 0.01 | 0.72 |  | -0.88 | 0.85 | 0.87 | -0.67 | 0.77 | 0.81 | 0.99^*^ | -0.05 | -0.56 | 0.67 | 0.58 | 0.75 | 0.28 | -0.40 | 0.02 |
| **Hem** | -0.49 | -0.93 | -0.88 |  | -0.94 | -0.98^*^ | 0.93 | -0.53 | -0.84 | -0.81 | -0.43 | 0.35 | -0.93 | -0.13 | -0.45 | -0.32 | 0.33 | -0.50 |
| **NDF** | 0.36 | 0.96^*^ | 0.85 | -0.94 |  | 0.99^*^ | -0.92 | 0.73 | 0.98^*^ | 0.75 | 0.30 | -0.63 | 0.92 | 0.25 | 0.64 | 0.62 | -0.01 | 0.42 |
| **ADF** | 0.42 | 0.96^*^ | 0.87 | -0.98^*^ | 0.99^*^ |  | -0.94 | 0.65 | 0.93 | 0.79 | 0.36 | -0.51 | 0.94 | 0.20 | 0.56 | 0.50 | -0.15 | 0.47 |
| **RA** | -0.69 | -0.99^**^ | -0.67 | 0.93 | -0.92 | -0.94 |  | -0.40 | -0.84 | -0.56 | -0.64 | 0.31 | -1.00^**^ | 0.14 | -0.29 | -0.47 | 0.07 | -0.73 |
| **RP** | -0.37 | 0.52 | 0.77 | -0.53 | 0.73 | 0.65 | -0.40 |  | 0.83 | 0.73 | -0.42 | -0.95^*^ | 0.40 | 0.80 | 0.99^*^ | 0.70 | 0.19 | -0.28 |
| **RF** | 0.21 | 0.90 | 0.81 | -0.84 | 0.98^*^ | 0.93 | -0.84 | 0.83 |  | 0.71 | 0.15 | -0.78 | 0.84 | 0.36 | 0.75 | 0.76 | 0.17 | 0.30 |
| **Rfi** | -0.08 | 0.60 | 0.99^*^ | -0.81 | 0.75 | 0.79 | -0.56 | 0.73 | 0.71 |  | -0.14 | -0.49 | 0.56 | 0.64 | 0.73 | 0.15 | -0.51 | -0.09 |
| **NfE** | 1.00^**^ | 0.55 | -0.05 | -0.43 | 0.30 | 0.36 | -0.64 | -0.42 | 0.15 | -0.14 |  | 0.43 | 0.64 | -0.84 | -0.54 | -0.01 | -0.08 | 0.98^*^ |
| **TS** | 0.39 | -0.43 | -0.56 | 0.35 | -0.63 | -0.51 | 0.31 | -0.95^*^ | -0.78 | -0.49 | 0.43 |  | -0.31 | -0.72 | -0.93 | -0.85 | -0.48 | 0.27 |
| **VS** | 0.69 | 0.99^**^ | 0.67 | -0.93 | 0.92 | 0.94 | -1.00^**^ | 0.40 | 0.84 | 0.56 | 0.64 | -0.31 |  | -0.14 | 0.29 | 0.47 | -0.07 | 0.73 |
| **N** | -0.80 | -0.03 | 0.58 | -0.13 | 0.25 | 0.20 | 0.14 | 0.80 | 0.36 | 0.64 | -0.84 | -0.72 | -0.14 |  | 0.88 | 0.24 | -0.07 | -0.77 |
| **TKN** | -0.49 | 0.40 | 0.75 | -0.45 | 0.64 | 0.56 | -0.29 | 0.99^*^ | 0.75 | 0.73 | -0.54 | -0.93 | 0.29 | 0.88 |  | 0.62 | 0.14 | -0.41 |
| **BD** | 0.01 | 0.56 | 0.28 | -0.32 | 0.62 | 0.50 | -0.47 | 0.70 | 0.76 | 0.15 | -0.01 | -0.85 | 0.47 | 0.24 | 0.62 |  | 0.76 | 0.17 |
| **P1** | -0.10 | 0.00 | -0.40 | 0.33 | -0.01 | -0.15 | 0.07 | 0.19 | 0.17 | -0.51 | -0.08 | -0.48 | -0.07 | -0.07 | 0.14 | 0.76 |  | 0.04 |

**Table S1.** Pearson’s correlation coefficients of the physio-chemical charactieristics of chicken manure with material permeability. Statistically significant values are indicated by symbols: **P < 0.01; *P < 0.05. NFC, non- fiber carbohydrates; Cel, cellulose; Lig, ligning; Hem, hemicellulose; NDF, neutral detergent fiber; ADF, acid detergent fiber; RA, raw ash; RP, raw rotein; RF, raw fat; Rfi, raw fiber; NfE, nitrogen free extract; TS, total solids; VS, volatile solids; N, ammonium nitrogen (NH_4_-N); TKN, total Kjeldahl nitrogen; BD, bulk density; P1 permeability (without compaction); P4, permeability (permeability at 3.5 m simulated material heap height)
